# Exercise-induced ‘browning’ of adipose tissues

**DOI:** 10.1016/j.metabol.2017.11.009

**Published:** 2018-04

**Authors:** Peter Aldiss, James Betts, Craig Sale, Mark Pope, Helen Budge, Michael E. Symonds

**Affiliations:** a The Early Life Research Unit, Division of Child Health, Obstetrics and Gynaecology, University of Nottingham, Nottingham NG7 2UH, UK; b Department for Health, University of Bath, Bath, BA2 7AY, UK; c Musculoskeletal Physiology Research Group, Sport, Health and Performance Enhancement Research Centre, School of Science and Technology, Nottingham Trent University, Nottingham, UK; d Nottingham Digestive Disease Centre and Biomedical Research Centre School of Medicine, University Hospital, University of Nottingham, Nottingham, UK, NG7 2UH

**Keywords:** Exercise, Brown adipose tissue, Browning, White adipose tissue

## Abstract

Global rates of obesity continue to rise and are necessarily the consequence of a long-term imbalance between energy intake and energy expenditure. This is the result of an expansion of adipose tissue due to both the hypertrophy of existing adipocytes and hyperplasia of adipocyte pre-cursors. Exercise elicits numerous physiological benefits on adipose tissue, which are likely to contribute to the associated cardiometabolic benefits. More recently it has been demonstrated that exercise, through a range of mechanisms, induces a phenotypic switch in adipose tissue from energy storing white adipocytes to thermogenic beige adipocytes. This has generated the hypothesis that the process of adipocyte ‘browning’ may partially underlie the improved cardiometabolic health in physically active populations. Interestingly, ‘browning’ also occurs in response to various stressors and could represent an adaptive response. In the context of exercise, it is not clear whether the appearance of beige adipocytes is metabolically beneficial or whether they occur as a transient adaptive process to exercise-induced stresses. The present review discusses the various mechanisms (*e.g*. fatty acid oxidation during exercise, decreased thermal insulation, stressors and angiogenesis) by which the exercise-induced ‘browning’ process may occur.

## Graphical abstract

Mechanisms of exercise-induced browning. Discussed in detail in-text.

**Figure:**
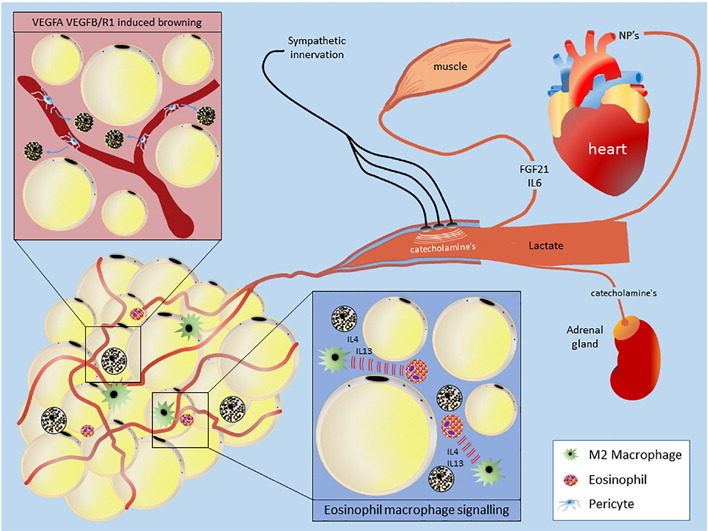

## 1 White, Brown and ‘Beige’ Adipose Tissue

Adipose tissue typically accounts for ~ 20–28% of total body mass in lean humans, with variance largely due to biological sex, and in the obese state can account for ~ 80% of body weight [1]. It is well understood that adipose tissues vary in their function and are dependent on both the type and anatomical location of the depot. Subcutaneous adipose tissue (ScAT), located under the skin, accounts for the majority of total white adipose tissue (WAT) in humans [2]. Visceral adipose tissue (VAT), located around the kidneys (perirenal), intestines (mesenteric and omental), vasculature (perivascular) and heart (epicardial/paracardial) normally accounts for much less but poses a far greater cardiometabolic risk following expansion [3]. Far from being “merely” energy storage depots, adipose tissues are potentially the largest endocrine organ in the body and, by the secretion of numerous factors, can regulate a range of physiological functions including metabolic homeostasis, appetite, angiogenesis, immunity and the cardiovascular system [4].

Conversely, brown adipose tissue (BAT) is located primarily in the supraclavicular region but also in smaller amounts around the kidneys, vasculature and heart [5]. BAT is responsible for adaptive thermogenesis, which preserves homeostasis in response to a thermal stimulus (*e.g.* temperature and/or energy balance); for example by uncoupling oxidative metabolism from ATP production in favour of heat production *via* uncoupling protein 1 (UCP1) [6]. The re-discovery of this tissue in adult humans [7] and its association with multiple parameters of metabolic health [8], [9], [10] and cardiovascular events [11] has led to huge interest in the potential to activate this tissue to combat obesity-associated cardiometabolic disease. More recently it has been shown that, following a stimulus (*e.g.* exercise), brown-like UCP1 + cells termed ‘beige’ adipocytes appear interspersed in what were previously classical white depots [12], [13]. It has therefore been suggested that these beige adipocytes: a) are distinct in origin, deriving from a lineage not shared with brown or white adipocytes; b) appear due to the trans-differentiation of pre-existing white adipocytes; or c) arise from a combination of the above [14]. It is important to note, however, that the molecular signature of both brown and beige adipocytes differs between small animals and humans [15]. In rodents, brown and beige adipocytes are anatomically distinct and clearly distinguishable whereas in humans current evidence suggests BAT is a heterogeneous depot expressing markers of both brown and beige adipocytes [16], [17]. As such, for the purpose of this review we will use the term ‘browning’ to describe the appearance of UCP1 + adipocytes. Species differences in molecular signature aside, the ‘browning’ of white adipose tissues has become an attractive therapeutic target due to mounting evidence suggesting that these ‘beige’ cells are metabolically active, contributing to both thermogenesis and metabolic health [17], [18].

Exercise and physical activity play a key role in modulating many parameters of cardiometabolic health, eliciting several benefits on adipose tissues [19]. A reduction in adipocyte size can be seen with exercise training [20], although this effect seems to be sex-specific as females undergoing the same training regime as men exhibited no changes in body mass or adipocyte size [21]. Furthermore, exercise training elicits improvements in adipose tissue inflammation [22], vascularity [23] and mitochondrial biogenesis [24], increasing the supply of oxygen and nutrients to the tissue and improving their oxidative capacity. More recently it has emerged that factors produced during exercise by skeletal muscle, adipose tissue and potentially the liver act to induce the ‘browning’ of WAT in an endocrine and/or paracrine manner. Of these, irisin, a PPARγ coactivator-1α (PGC1-α) dependent myokine [25], has generated the most interest as a browning agent, although there is major controversy surrounding the results and validity of this purported myokine [26]. Numerous other factors that promote ‘browning’ are also altered during or following exercise, such as interleukin-6 [27], B-aminoisobutyric acid [28], meteorin-like [29], fibroblast growth factor-21 [30], natriuretic peptides (NP's) [31], [32], [33] and lactate [34], leading many to suggest that the long-lasting metabolic effects of exercise may be due to the ‘browning’ of white adipose tissues. However, many of these indices have only been validated *in-vitro* or in animal models and as such their role in ‘browning’ human WAT are currently unknown.

## 2 Regulation of Adipose Tissue Metabolism During Exercise

Despite the therapeutic potential of thermogenic ‘beige’ cells, it is not entirely clear why they appear during exercise, a time of heat production [35]. It has been suggested, however, that following the lipolytic response to exercise and subsequent increase in circulating non-esterified fatty acids (NEFA) there is a need for other areas of oxidation and that the browning of white adipocytes provides these sites in order to maintain NEFA flux [36]. Adipose tissue metabolism during exercise is regulated by multiple factors released both centrally and peripherally to modulate the rate of NEFA uptake and release. The major endocrine mechanism is increased plasma epinephrine which, acting through cyclic AMP (cAMP) and protein kinase A (PKA), phosphorylates adipose triglyceride lipase (ATGL) to stimulate lipolysis and maintain NEFA supply during exercise, a response that is abolished following the blockade of B-adrenoreceptors with propanalol [37], [38]. Other lipolytic factors include norepinephrine (NE), glucagon, cortisol and NP's [37]. NE only contributes marginally to exercise-induced lipolysis [38], whilst glucagon and cortisol concentrations increase later in exercise and bind to stimulatory GTP-binding protein to activate ATGL through cAMP and PKA [37]. NP's are increased during exercise of moderate intensity and stimulate lipolysis through the activation of cGMP-dependant protein kinase I (cGKI) and subsequent phosphorylation of perilipin 1 and hormone-sensitive lipase (HSL) [39]. Altered concentrations of adenosine and insulin act against these signals to regulate lipolysis and prevent excess release of NEFA, which is apparent when the typical exercise-induced reduction in insulin is absent [40]. Post-exercise, lipolysis stabilises but remains higher compared to rest for up to 24 h, thus even a single bout of exercise can influence energy expenditure/balance over the next day [41] and modulate insulin sensitivity for up to 48 h [42]. Whilst exercise elicits substantial alterations in adipose tissue metabolism, whether the ‘browning’ that occurs following exercise training occurs during an acute bout of exercise to facilitate the oxidation of excess NEFA as postulated by Virtanen [36] remains to be determined. Intriguingly, two recent groups have demonstrated that BAT lipolysis is not essential for cold-induced thermogenesis whereas WAT lipolysis is crucial, fuelling thermogenesis during fasting [43], [44]. It would be interesting to know, in the context of the hypothesis by Virtanen [36], whether WAT lipolysis is also essential for the ‘browning’ seen following exercise.

## 3 Is Exercise Induced ‘Browning’ a Consequence of Reduced Adiposity?

Regular exercise can improve body composition, including reduced adiposity and/or increased muscle mass. A particularly interesting recent finding was that the phenotypic switch towards thermogenic brown cells shown following exercise in rats primarily occurred in subcutaneous depots, which was concomitant with alterations in BAT morphology (*i.e.* whitening) and a decreased thermogenic capacity [45]. Exercise training reduced adiposity but the appearance of multilocular adipocytes, thermogenic genes and increase in oxidative capacity were only evident in the inguinal depot (visceral depots being resistant), suggesting depot-specificity. These inter-depot differences, also highlighted by De Jong *et al.* [46], are of particular interest as exercise does not induce browning in human WAT biopsies [47] and suggests a single biopsy is not an appropriate method to detect browning in humans (at least until we better understand how this process is regulated across both a single depot and between depots). Whilst challenging, future human studies should be designed to examine the response to exercise both at multiple sites in the same adipose depot and also across different depots.

The ‘whitening’ of BAT and reduced thermogenic capacity following exercise training was suggested to be a counter-regulatory mechanism whereby the ability of BAT to expend energy is reduced during times of increased energy expenditure [48], [49], [50]. However, the discovery that exercise induces browning of subcutaneous depots whilst simultaneously increasing lipid content in BAT has led to suggestions for other roles of exercise induced ‘browning’. It is now postulated, in rodents, that a reduction in BAT occurs in response to regular increases in core body temperature, whilst the browning of subcutaneous WAT occurs due to a reduction in total mass and thus the degree of insulation conferred by this depot [45], [51]. Whilst this theory is consistent with multiple findings that high-fat feeding and resultant excess subcutaneous adiposity decreases UCP1 content [52], recent evidence in mice suggests that adipose tissue does not confer any insulatory properties [53]. However, given that rodents are typically cold-exposed throughout life [54], the ‘browning’ seen in subcutaneous adipose tissues following exercise could be an adaptive mechanism to increase the thermal potential of reduced adiposity, thus offsetting any increased sensation of peripheral cold. Interestingly, recent work by Peppler and colleagues demonstrated that removal of IWAT by lipectomy did not attenuate the metabolic benefits of wheel-running in mice and that a compensatory browning was not shown in epididymal WAT despite the reduction in adiposity [55]. However, compensatory browning in the face of reduced adiposity may be limited to ScAT. Had other ScAT depots, or in fact dermal AT been collected an induction of thermogenic genes may have been present. Whilst the authors note the limited clinical relevance of their model it suggests the importance of exercise-induced browning of IWAT is negligible and is of particular relevance given that humans have not been shown to exhibit ‘browning’ following exercise but still exhibit numerous metabolic benefits [56], [57], [58]. Whilst human WAT biopsies may not be the most effective way to detect ‘browning’ due to the limited amount of tissue relative to depot mass, exercise does reduce BAT mass and activity in athletic humans [47], [59]. This is consistent with rodent data and the notion that the thermogenic capacity of BAT may be reduced in the presence of regular increases in core body temperature. Given the inverse relationship between BAT mass and lean mass [23], it is possible that the recruitment of a large muscle mass during exercise training negates the need for BAT, given the potentially superior capacity for muscle to generate heat [60].

## 4 ‘Browning’ as an Adaptive Mechanism to Exercise-Induced Stress?

There is a growing consensus that the ‘browning’ of adipose tissues occurs as an adaptive mechanism to certain stresses, aside from the atypical stress of a reduced ambient temperature. This stress-induced ‘browning’ has been shown in numerous clinical populations including those suffering burn injuries and pheocromocytoma [13], [61]. The hypermetabolic state induced by burn injury is derived from a chronic increase in circulating catecholamines and IL-6 which induce the remodelling of subcutaneous adipose tissue [13], [62]. This chronic stress increases mitochondrial mass in ScAT, expression of thermogenic markers such as UCP1 and PGC1a in addition to whole body energy expenditure [63]. These factors that are thought to play a major role in the hypermetabolism evident in cancer patients, contributing to the cachexic state [64], [65]. Regular physical exercise induces multiple physiological stressors such as regular chronic sympathetic activation leading to increases in catecholamine secretion and IL-6 after only 6 min of exercise [66], whilst 25-fold increases in IL-6 are seen during longer running protocols in humans and are maintained 2.5 h post-exercise [67]. Whether the effects of IL-6 on adipose tissue are due to its pro- or anti-inflammatory properties or due to the synergistic immune response following exercise which includes alterations in IL-1ra and IL-10 [68] for instance is unknown.

Rodent studies suggest that the metabolic benefits of both BAT and the exercise-induced browning of ScAT are dependent on the presence of IL-6, with no effect in IL-6 k/o mice; again, whether this is simply an effect of IL-6 or due to the effect of its absence on other factors is unclear [69], [70]. Accumulating evidence suggests that ‘browning’ is associated with reduced adipose tissue inflammation, so the ‘browning’ seen with exercise could be an adaptive response to increased inflammatory markers such as IL-6 [22]. There are other factors indicating a role for browning as an adaptive mechanism to exercise-induced stresses. In particular, the recent finding that lactate administration induces browning, thus maintaining intracellular redox state [34]. Whilst lactate was not reported in response to exercise in that study, both the treatment of murine and human adipocytes with lactate and its exogenous administration *in-vivo* induced UCP1 in a redox state dependent manner [34]. Physical exercise is a source of free radicals, occurring primarily during strenuous or prolonged exercise and whilst these free radicals can, depending on the state of antioxidant defence, cause cellular damage at high levels they are also important signalling molecules [71]. In the context of exercise, the increase in free radicals could modulate changes in the intracellular redox state of WAT, with lactate being an evolutionary mechanism to modulate redox state by inducing the brown phenotype. BAT has an abundance of antioxidant enzymes, including UCP1 (which itself plays a key role in regulating ROS production) [72], [73] although, as discussed above, chronic exposure to a higher core temperature during exercise training may downregulate its activity. As such, ‘browning’ in other depots may be a compensatory mechanism for a reduction in BAT mitochondrial function.

Another mechanism by which “browning” could occur is through the increase in NP secreted from cardiac tissues in response to the increased stress and demands placed on the cardiac system during exercise [31]. Whilst NP induce browning in subcutaneous adipose tissues [31], it is feasible that their prime target is the local adipose tissues around heart and vasculature that modulate both myocardial [74] and vascular [75] redox state, in order to attenuate excess oxidative stress within the heart and vasculature.

## 5 Does Exercise-Induced Angiogenesis Modulate the Expression of Beige Cells?

An increase in angiogenesis and vascularisation of tissues and organs, with raised oxygenation, blood flow and nutrient delivery is one of the mechanisms through which exercise improves cardiometabolic health [76]. Unlike classical BAT which shares origins with skeletal muscle, beige adipocytes were originally shown to originate from vascular smooth muscle cells and subsequently it was demonstrated that most, if not all beige adipocytes are derived from the vascular niche [77], [78]. This is particularly interesting given that browning occurs in a distinct region of the inguinal depot along the main vasculature and suggests that angiogenesis and/or the proliferation of vascular smooth muscle cells into beige adipocytes may be key to this regional browning effect [79]. Further evidence to support a role of exercise-induced angiogenic mechanisms in the induction of the beige phenotypes comes from data demonstrating that adipose specific over-expression of vascular endothelial growth factor (VEGF; a key regulator of angiogenesis) reproduces the browning that occurs in an enriched environment, effects which were negated with adipose specific VEGF k/o and VEGF blockade [80]. This suggests that VEGF and related angiogenic mechanisms are essential for the browning effect induced by exercise.

More recently it has been shown that expansion of the adipose vasculature by another member of the VEGF family, VEGFB (plus its receptor VEGFR1), prevents the occurrence of several aspects of obesity-induced metabolic dysfunction concomitant with increased vascularisation and thermogenic markers in WAT [81]. In the obese state the dysfunction of adipose tissues and the ‘whitening’ of BAT stems from the adipocytes becoming hypertrophic and outstripping the vasculature to become hypoxic [82], [83]. Thus it is likely that the restoration of the vascular supply to these tissues can induce or restore the brown phenotype and, in the case of exercise, the induction of angiogenic genes will promote the vascular supply in WAT to induce ‘browning’. Whether ‘browning’ is an essential end-product of this process or a simple by-product of an increased vascularisation and differentiation of pre-cursor cells from the vascular niche remains to be determined. Furthermore, it is worth noting the presence, location and relevance of the lymph node in relation to the regionalisation of ‘browning’ demonstrated by Barreau *et al.* [79].

Exercise training triggers multiple complex changes in immune function [84] and emerging evidence suggests that innate immune cells control brown and beige adipogenesis [85]. Meteorin-like is produced following resistance training *via* the induction of the PGC1α isoform PGC1α 4 and induces the browning of adipose tissues by stimulating IL-4 and IL-13 secretion from eosinophils and the activation of alternate M2 macrophages [29]. Whilst the role of catecholamine production from macrophages has recently been questioned, the use of bone-marrow derived macrophages and not tissue resident macrophages leaves such questions unanswered, particularly as the latter may play a local role in immune-adipose crosstalk. Just as UCP2 regulates immune cell metabolism, differentiation and survival, it is feasible UCP1 may play a similar role in adipose tissue [86], [87]. Finally, immune cells produced post-exercise from the lymph node may have a local effect on neighbouring adipocytes leading to the region specific ‘browning’ seen by others [79].

## 6 Summary

Exercise, through various secreted factors and mechanisms induces a ‘browning’ of WAT. However, the physiological explanation for this process is unclear. From an evolutionary point of view the preservation of energy stores is vital. Why then would exercise cause the appearance of thermogenic adipocytes that can increase energy expenditure? It is plausible that our lean and active ancestors naturally possessed large quantities of brown or beige adipocytes. Our subsequent transition to a modern, largely sedentary population has meant these adipocytes have undergone a ‘whitening’ and the ‘browning’ that occurs in response to exercise is actually a transition back to their natural state. Despite a lack of understanding as to why exercise should elicit these effects on adipose tissue, it is worth noting Orgel's second law: “Evolution is cleverer than you are”. Based on this rule there is likely a valid physiological explanation(s) for the ‘browning’ process that is yet to be discovered.

Whilst exercise induces a brown phenotype in rodent ScAT, it is not yet clear whether exercise elicits similar effects in humans though the available data suggests that, similar to rodents, exercise downregulates both the mass and activity of human BAT. Whether this is compensated for by browning of WAT or is due to the significant increases in muscle mass and therefore greater capacity for shivering thermogenesis remains to be established.

At present both rodent and human evidence is based on either sedentary or exercise-trained populations, with little attention given to the role of physical activity *per se*. Whether regular physical activity rather than exercise training potentiates any thermogenic effects on adipose tissues is unknown but, considering the effects of regular physical activity on adiposity and cardiometabolic health, is an area that merits investigation. Studies investigating regular physical activity would also not be confounded by increases in core body temperature and reductions in adiposity, so would rule out these hypothetical mechanisms.
